# Cardiorespiratory fitness levels and body mass index of pre-adolescent children and older adults during the COVID-19 pandemic

**DOI:** 10.3389/fpubh.2022.1052389

**Published:** 2023-01-17

**Authors:** Lauren B. Raine, Kirk I. Erickson, George Grove, Jennifer N. H. Watrous, Katherine McDonald, Chaeryon Kang, John M. Jakicic, Daniel E. Forman, Arthur F. Kramer, Jeffrey M. Burns, Eric D. Vidoni, Edward McAuley, Charles H. Hillman

**Affiliations:** ^1^Department of Physical Therapy, Movement, and Rehabilitation Sciences, Northeastern University, Boston, MA, United States; ^2^Department of Psychology, University of Pittsburgh, Pittsburgh, PA, United States; ^3^PROFITH “PROmoting FITness and Health Through Physical Activity” Research Group, Department of Physical and Sports Education, Faculty of Sport Sciences, Sport and Health University Research Institute (iMUDS), University of Granada, Granada, Spain; ^4^Research Institute, AdventHealth, Orlando, FL, United States; ^5^Department of Psychology, Oklahoma State University, Stillwater, OK, United States; ^6^Department of Psychology, Northeastern University, Boston, MA, United States; ^7^Department of Biostatistics, University of Pittsburgh, Pittsburgh, PA, United States; ^8^Department of Internal Medicine, University of Kansas Medical Center, Kansas City, KS, United States; ^9^Department of Medicine and Veterans Affairs Pittsburgh Healthcare System, Geriatrics, Research, Education and Clinical Care (GRECC), University of Pittsburgh, Pittsburgh, PA, United States; ^10^Beckman Institute, University of Illinois, Urbana, IL, United States; ^11^Alzheimer's Disease Research Center, University of Kansas Medical Center Fairway, Fairway, KS, United States; ^12^Department of Kinesiology and Community Health, University of Illinois Urbana Champaign, Champaign, IL, United States

**Keywords:** COVID-19, cardiorespiratory fitness, body mass index (BMI), older adults, children

## Abstract

**Introduction:**

The social and behavioral effects of the COVID-19 pandemic have impacted the health and physiology of most people, including those never diagnosed with COVID-19. While the impact of the pandemic has been felt across the lifespan, its effects on cardiorespiratory fitness (commonly considered a reflection of total body health) of older adults and children may be particularly profound due to social distancing and stay-at-home advisories, as well as the closure of sport facilities and non-essential businesses. The objective of this investigation was to leverage baseline data from two ongoing clinical trials to determine if cardiorespiratory fitness and body mass index were different during COVID-19 relative to before COVID-19 in older adults and children.

**Methods:**

Healthy older individuals (*N* = 593; 65–80 years) and 200 typically developing children (8–10 years) completed a graded maximal exercise test and had their height and weight measured.

**Results:**

Results revealed that older adults and children tested during COVID-19 had significantly lower cardiorespiratory fitness levels than those tested before COVID-19 shutdowns (older adults: 30% lower; children: 53% lower; *p's* ≤ 0.001). In addition, older adults and children tested during COVID-19 had significantly higher BMI (older adults: 31.34 ± 0.57 kg/m^2^, *p* = 0.004; children: 19.27 ± 0.44 kg/m^2^, *p* = 0.05) than those tested before COVID-19 shutdowns (older adults: 29.51 ± 0.26 kg/m^2^, children: 18.13 ± 0.35 kg/m^2^). However, these differences in BMI did not remain significant when controlling for cardiorespiratory fitness.

**Discussion:**

Results from this investigation indicate that the COVID-19 pandemic, and behavior changes taken to reduce potential exposure, may have led to lower cardiorespiratory fitness levels in older adults and children, as well as higher body mass index. These findings provide relevant public health information as lower cardiorespiratory fitness levels and higher body mass indexes recorded during the pandemic could have far-reaching and protracted health consequences. Public health guidance is needed to encourage physical activity to maintain cardiorespiratory fitness and healthy body composition.

**Clinical trial registration:**

Older adults: https://clinicaltrials.gov/ct2/show/NCT02875301, identifier: NCT02875301; Children: https://clinicaltrials.gov/ct2/show/NCT03592238, identifier: NCT03592238.

## Introduction

On March 11th, 2020, the World Health Organization declared the novel coronavirus disease 2019 (COVID-19) a global pandemic (1). To prevent the spread of COVID-19, a variety of responses were implemented including stay-at-home advisories, social distancing, and the closure of non-essential businesses, including fitness centers, parks/playgrounds, and schools. One undesired consequence of the COVID-19 shutdowns, stay-at-home advisories, and behavior changes out of concern for the virus was a significant decline in physical activity (PA) (2–4), which was already below recommended levels (5, 6) prior to the onset of the COVID-19 pandemic. Global data demonstrates that PA decreased by >20% compared to before the COVID-19 stay at home advisories, with reductions particularly pronounced in older adults (7). Within 30 days of when COVID-19 was declared a global pandemic, there was a 27.3% worldwide decrease in average daily steps (8). Such findings could have serious public health implications because physical inactivity is a leading cause of cardiovascular disease, Type 2 diabetes, obesity, and premature mortality (5). These chronic diseases typically do not emerge before adulthood, but can be provoked by inactive behaviors (9). Chronic diseases are also linked to sedentary behaviors or activities that take place in a seated or reclined positions and require little energy expenditure. In particular, higher amounts of sedentary behaviors are related to an increased risk of mortality, cardiovascular disease, Type 2 diabetes, and some cancers (10). PA influences an individual's risk of disease because it modifies various physiological mediators of disease outcomes including cardiorespiratory fitness (CRF). Coincident with a decrease in PA, CRF levels have also decreased in recent years in adults between 18 and 59 years old (11). This decrease in CRF, which may be influenced by the concurrent decrease in PA, is of concern because CRF is a predictor of all-cause mortality, cardiovascular disease, Alzheimer's disease, and other diseases even after adjusting for risk factors including hypertension, smoking, and obesity (12). CRF is also related to risk for some cancers, kidney disease, Type 2 diabetes, neurologic complications, and bone health (13–15).

The harmful physiological and behavioral consequences of the COVID-19 pandemic are well-documented, even in individuals undiagnosed with the COVID-19 virus (16–18). While the pandemic has affected individuals across the lifespan, CRF of older adults and children may be particularly susceptible to changes due to the social distancing and stay-at-home advisories. Older adults are particularly vulnerable to rapid deconditioning of the musculoskeletal system with decreased PA, the effects of which are hard to reverse (19). While children were not particularly vulnerable to adverse outcomes from COVID-19 infection, children did experience substantial changes to daily routines and lifestyles, which typically provides a source of resilience during challenging times. With the shutdown of most schools, the primary daily structure of children's lives was substantially altered. In addition, for many children, sports and activities were canceled; and parks, playgrounds, and recreation centers were closed (20). One inadvertent consequence of closing of these facilities appears to also be an increase in sedentary behaviors among children. The most common sedentary behaviors that parents reported in their children was watching tv/movies/videos as well as sitting (20). Some research suggests that up 91% of parents perceived an increase in the time their child spent sitting during the COVID-19 shutdown (21). In addition to the physiological consequences of the pandemic (for example, changes in CRF) on older adults and children, the pandemic may also have had an extensive psychological impact. It is likely that both the psychological and physiological consequences of the pandemic (i.e., CRF) could have persistent and long lasting effects (22, 23).

In addition to changes in PA, there were also changes in other behaviors and subsequent health conditions over the COVID-19 pandemic. For example, compared to pre-pandemic levels, the rate of increase in body mass index (BMI) nearly doubled in over 400,000 American children and adolescents between 2 and 19 years old during the pandemic, with the greatest increases in children with overweight or obesity (24). In addition, children ages 5–11 years had the greatest acceleration in weight trajectories (24). Thus, it is not surprising that studies have found that the number of children with overweight, obesity, or morbid obesity increased significantly over the pandemic (24–27), a worrying increase over a short period. These increases in weight and BMI have co-occurred alongside a decrease in exercise and PA in children (28, 29).

Changes in weight and BMI in adults during the COVID-19 pandemic have been mixed. In over 800 participants between 16 and 70 years old from Jiangsu, China individuals, on average, gained weight after only 2 months of the COVID-19 lockdown, with some individuals gaining almost 11 kgs (30). In contrast, a study of 4,400 older adults (65 years and older) in Northern Italy did not find a significant change in weight or BMI (31). In the United States, studies suggest that somewhere between 27.5 and 40% of individuals gained weight (32, 33).

There are a variety of reasons why adults may have gained weight during the COVID-19 pandemic. For example, research suggests that during the COVID-19 shutdown, people spent significantly more time sitting every day (21, 34). In addition, according to 30 million FitBit users, there was a significant decline in step counts (week ending on March 22, 2020 compared to same week in 2019) (35). Together, these changes may have long term adverse consequences on chronic disease risk and health, and could subsequently impact the health of the population in the future (25). In addition to changes in PA, there were also changes in dietary patterns during the COVID-19 pandemic (33). For example, in over 5,000 individuals there was a decrease in meals consumed from restaurants and pre-prepared; increased consumption of sweets and sugar-sweetened beverages; and increased unhealthy snacking (32).

Most of the previous data is based on self-reported surveys completed by middle aged adults. These surveys ask participants to self-report changes in weight and health behaviors. While these findings are important, it is critical to analyze the effects of the COVID-19 pandemic shutdowns using more precise lab-based measures. Children and older adults were both highly susceptible to the unintended consequences of the pandemic. Thus, this investigation examined differences in CRF and BMI in samples of older adults and children to better understand the impact of the pandemic on these important physiological outcomes. We utilized baseline data from two ongoing randomized clinical trials of exercise to examine whether individuals that underwent CRF and BMI testing before the COVID-19 shutdown differed in CRF levels and BMI from individuals tested after the COVID-19 shutdown. Specifically, we predicted that individuals examined following the start of the COVID-19 stay-at-home advisory would have lower CRF and higher BMI compared to those individuals collected before the COVID-19 stay-at-home advisory. Furthermore, we speculated that such differences might have been exacerbated with the progression of the pandemic such that the number of days since the onset of the COVID-19 stay at home advisory would exhibit increased differences from pre-pandemic levels.

## Materials and methods

We leveraged baseline data from two ongoing randomized clinical trials (IGNITE: Investigating Gains in Neurocognition in an Intervention Trial of Exercise, NCT02875301; SNEACY: Sympathetic Nervous System and Exercise Affects Cognition in Youth, NCT03592238) that spanned pre-pandemic (September 2017–March 13, 2020) through pandemic periods (August 5, 2020–October 2021) to examine differences in average CRF and BMI levels in groups before and following the start of the COVID-19 stay-at-home advisory. The two trials include relatively healthy older individuals (65–80 years, 23% non-white) from three different geographical sites in the U.S. (Pittsburgh, PA; Boston, MA; Kansas City, KS), and typically developing children (8–10-years, 42% non-white) from Boston, MA, see Table 1 for participant demographics. Recruitment strategies, eligibility, testing protocols, BMI measurements, and CRF protocols were published before the start of the pandemic (36, 37) and did not change over the course of the pandemic. All participants provided written informed consent (or assent in the case of children) in accordance with the Institutional Review Board at their testing site. Briefly, for the trial in older adults, criteria included cognitively normal individuals who were relatively inactive but could still safely participate in moderate intensity exercise. Cognitive health was assessed using the Telephone Interview for Cognitive Status (TICS), a widely used instrument that is highly correlated with scores on the Mini-Mental State Examination (MMSE), and participants had to have a score of 26 or greater to be included. Participants were screened by clinical neuropsychologists for dementia or mild cognitive impairment (MCI) (37). Older adults were also cleared by their Primary Care Physician (PCP) to engage in exercise and were free from serious neurological conditions including Multiple Sclerosis, Parkinson's Disease, Dementia, traumatic brain injury, seizures, or any prior strokes, as well as schizophrenia, other psychotic disorders, or bipolar disorder. All older adults who expressed an interest in the study and met the selection criteria were invited to participate.

**Table 1:** Participant demographics.

	**Pre-COVID** **(Mean ±SE)**	**Co-COVID** **(Mean ±SE)**
**Older adults**		
*N*, %female	493, 71.6%	100, 66.0%
Age (years)	69.63 ± 0.16 (65–80)	70.71 ± 0.42 (65–80)
**Children**		
*N*, %female	122, 49.2%	78, 30.8%
Age (years)	9.74 ± 0.06 (8–11)	10.09 ± 0.10 (8–11.4)

For the study with children, the eligibility criteria were designed to recruit participants who were typically developing, generally healthy, and able to safely exercise. Children had to be capable of performing a maximal exercise test; have normal IQ or above (as assessed using the Kaufman Brief Intelligence Test-2); no diagnosis of cognitive or physical disability (ADHD, uncontrolled asthma, epilepsy, chronic kidney disease, able to walk unassisted; free of any anti-psychotic, anti-depressant, anti-anxiety, ADHD/ADD medication; able to speak and read English). All children who expressed an interest in the study and met the selection criteria were invited to participate.

### Graded exercise testing

CRF was measured at baseline, before beginning either trial, by conducting a graded maximal exercise test (38, 39) to determine CRF levels *via* VO_2_max following modified Balke protocols on a motor-driven treadmill (38). For both older adults and children, ACSM guidelines were used to conduct the exercise test, during which exhaled air was collected and analyzed. Greater details are provided below. Relative peak oxygen consumption is expressed in ml/kg/min, and normative data, accounting for age and sex, were used to calculate percentiles (VO_2_max%) (40) for each participant.

#### Older adults

Older adults completed a VO_2_max test using either a COSMED Quark CPET OMNIA (Concord, California) or Parvo Medics True 2,400 system. First, a brief warm-up session, including resting blood-pressure measurements and resting electrocardiogram (ECG) review were completed. Next, the participant walked on a treadmill at a constant speed (1.5–3.5 mph—based on participant ability and exercise physiologist guidance). The intensity was increased every 2 min with a 2% increase in incline. Heart rate (HR) was monitored continuously *via* a 12 lead ECG. Blood pressure measurements and Rating of Perceived Exertion (RPE) were taken every 2 min. When the participant reached the endpoint of the exercise test (symptom limitation and/or volitional exhaustion), they completed a 4 min active cool-down followed by a 4-min seated cooldown. Maximal exertion was determined when three of the four following criteria were met: (1) Plateau in VO_2_ between two or more workloads (increase < 0.15 L/min or 2.0 mL/kg/min during the last minute of corresponding work-loads). (2) Respiratory Exchange Ratio (RER) equal to or >1.10; (3) HR within 10 beats of age predicted maximal HR (220-age) (41); (4) RPE > 17 (42).

#### Children

Children completed a VO_2_max test on a motorized treadmill (Treadmill: Trackmaster TMX428; Metabolic cart: COSMED Quark CPET OMNIA, Concord, California). Participants completed a brief walking warmup and then ran at a constant speed with incremental intensity increases through grade inclines of 2.5% every 2 min until volitional fatigue. The speed was determined based on the child's height, running stride, and overall ability of the child. Participants wore a HR monitor during the test to determine maximal HR. RPE was assessed every 2 min using the children's OMNI scale (42). Maximal exertion was determined when three of the four following criteria were met: (1) Plateau in VO_2_ between two or more workloads (increase < 0.15 L/min or 2.0 mL/kg/min during the last minute of corresponding work-loads). (2) Respiratory Exchange Ratio (RER) ≥ 1.0 (43); (3) a peak HR ≥ 185 bpm and a HR plateau (41, 44); (4) RPE on the children's OMNI scale of perceived exertion ≥ 8 (42).

### BMI measurement

Weight, measured in kilograms (kg), and height, measured in meters (m), were assessed while participants were barefoot and wearing lightweight clothing. BMI was calculated as kg/m^2^. Scales and stadiometers were calibrated at each site. For children only, BMI percentile (BMI%) was calculated based on CDC growth charts (ages 2–19 y), accounting for age and sex (45). Such percentiles are not available in adult populations.

The number of days since the start of the COVID-19 stay-at-home advisory was calculated using March 13th, 2020 as the critical date with any testing occurring before this date labeled as pre-COVID and any test happening after this date as co-COVID. This date was used because it was the last day that the research sites were open before the COVID-19 stay-at-home advisory occurred (two days after COVID-19 was declared a global pandemic).

### Statistical analyses

All statistical analyses were performed with SPSS 27 (IBM, Armonk, New York) using two-sided tests and the error rate across statistical tests, or the family-wise alpha threshold, for all tests set at *p* = 0.05. Each analysis was independent and no *post-hoc* comparisons were conducted. Levene's test for equality of variances was used to determine if the variances were different between pre- and co-COVID participants. If the Levene's test was significant, the statistics were based on equal variances not assumed, and if Levene's test was not significant, the statistics were based on equal variances assumed.

Using independent sample *t*-tests, differences in VO_2_max% were examined within each study, and by site (for older adults), comparing participants who completed testing before the stay-at-home advisory (referred to here as pre-COVID) from those that were tested following the start of the COVID-19 stay-at-home advisory (referred to here as co-COVID). In children, independent sample *t*-tests were used to examine descriptive differences in BMI%, comparing pre-COVID children and co-COVID children. Furthermore, within each age group, ANCOVAs controlling for age and sex were also conducted to compare VO_2_max and BMI between pre-COVID participants and co-COVID participants.

In addition, within each age group there was interest in investigating whether VO_2_max and BMI could be confounding factors in the analyses testing for associations with the other variable. Thus, ANCOVAs controlling for age, sex, and BMI were conducted to compare VO_2_max between pre-COVID participants and co-COVID participants. Subsequent ANCOVAs controlling for age, sex, and VO_2_max were conducted to compare BMI between pre- and co-COVID participants.

Finally, separate multiple hierarchical linear regression analyses were conducted within each age group to examine the impact of the number of days since the onset of the COVID-19 stay-at-home advisory on VO_2_max and BMI. In Step 1, the dependent variables were regressed on age and sex. To determine the unique contribution of each independent variable, the final step included the number of days since the onset of the COVID-19 stay-at-home advisory which was independently entered into Step 2. The change in *R*^2^-values between the two steps was used to judge the independent contribution of the number of days since the onset of the COVID-19 stay-at-home advisory for explaining the variance in the dependent variables of interest (VO_2_max and BMI) beyond that of demographic variables.

## Results

In both trials, none of the participants during co-COVID reported a prior diagnosis of COVID-19 at the time of the BMI or VO_2_max assessment. Skew and kurtosis were examined within each age group. For older adults, skew: VO_2_max = 0.37, BMI = 0.66; kurtosis: VO_2_max = −0.071, BMI = 0.29. For children, skew: VO_2_max = −0.13, BMI = 1.46; kurtosis: VO_2_max = 0.19, 2.49. These values for skew and kurtosis are between −2 and +2 and are therefore acceptable (46).

### Age

Children tested co-COVID (10.09 ± 0.10 yrs) were slightly older than those tested pre-COVID (9.74 ± 0.06 yrs), *p* = 0.003. Given that aerobic fitness increases during childhood (47, 48), this would suggest that if anything, children tested co-COVID should have had higher CRF. Older adults tested co-COVID (70.73 ± 0.42 yrs) were slightly but significantly older than those tested pre-COVID (69.63 ± 0.16 yrs), *p* = 0.003, see Table 1.

### Gender

In children, there was a greater percentage of males co-COVID (69.23%; 24 females, 54 males) compared to pre-COVID (50.82%; 60 females, 62 males), *p* = 0.01. In older adults, there was no difference in the percentage of males pre-COVID (28.40%; 353 females, 140 males) compared to co-COVID (34.0%; 66 females, 34 males), *p* = 0.26, see Table 1.

### Site

The ANCOVA analyses for VO_2_max% and BMI indicated that there was no main effect of testing site (*p's* = 0.43 and 0.83, respectively), and all three testing sites (Kansas City, Pittsburgh, Boston) showed statistically similar decreases in VO_2_max% and increases in BMI indicating uniformity of the effect across testing sites.

### Relative VO_2_max in older adults

**Figure 1:**
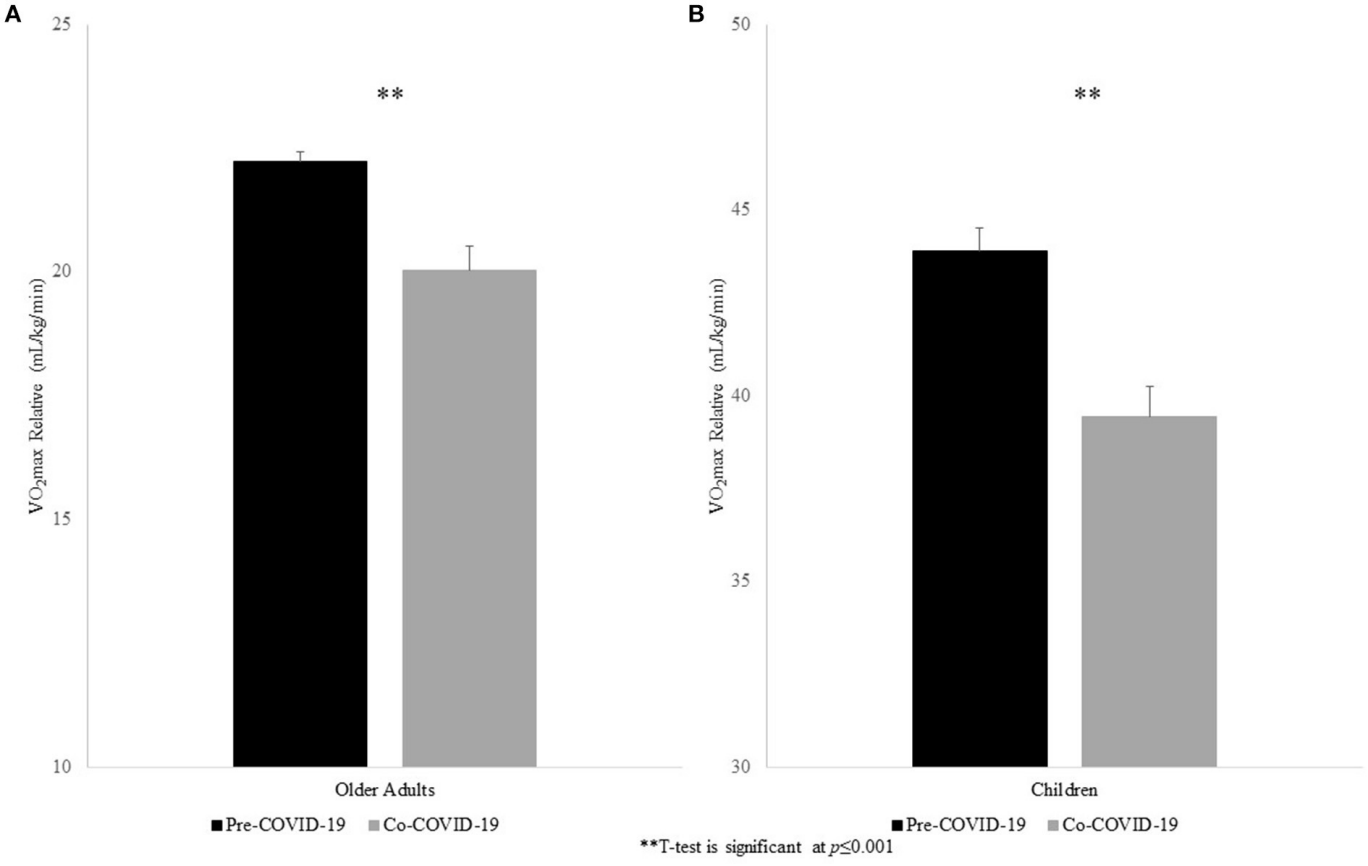
Relative VO_2_max (Means ± Standard Error) for Pre- and Co-COVID for older adults **(A)** and children **(B)**.

The ANCOVA for relative VO_2_max controlling for age and sex revealed differences between pre- and co-COVID older adults. Pre-COVID older adults achieved an average relative VO_2_max of 22.22 ± 0.21 ml/kg/min, which was significantly greater than their co-COVID counterparts (20.04 ± 0.47 ml/kg/min), *F*_(3, 589)_ = 17.96, *p* ≤ 0.001, η^2^ (effect size) = 0.03. This effect remained significant after also adjusting for BMI [*F*_(4, 587)_ = 9.74, *p* = 0.002, η^2^ = 0.02, see Figure 1].

### VO_2_max percentile in older adults

Pre-COVID older adults achieved an average VO_2_max% of 48.66 ± 1.25%, which was significantly greater than their co-COVID counterparts VO_2_max% 34.32 ± 2.59% (*t* = 4.78, *p* ≤ 0.001, CI: 8.67, 20.02). This equates to a 30% lower VO_2_max% in co-COVID older adult participants relative to pre-COVID.

### Relative VO_2_max in children

The ANCOVA for relative VO_2_max controlling for age and sex revealed differences between pre- and co-COVID children. Pre-COVID children achieved an average relative VO_2_max of 43.88 ± 0.65 ml/kg/min, which was significantly greater than their co-COVID counterparts (39.42 ± 0.82 ml/kg/min), *F*_(3, 196)_ = 17.55, *p* ≤ 0.001, η^2^ = 0.082, see Figure 1. This effect remained significant after also adjusting for BMI [*F*_(4, 195)_ = 13.64, *p* ≤ 0.001, η^2^ = 0.07].

### VO_2_max percentile in children

Similar effects were observed in children as were present in older adults. We found that pre-COVID children achieved a VO_2_max% of 36.66 ± 2.87%, which was significantly greater than the co-COVID participants VO_2_max% 17.08 ± 1.91% [*t*_(198)_ = 5.67, *p* ≤ 0.001, CI: 12.78, 26.40]. This is equivalent to a 53% lower VO_2_max% in co-COVID child participants relative to pre-COVID.

### BMI in older adults

**Figure 2:**
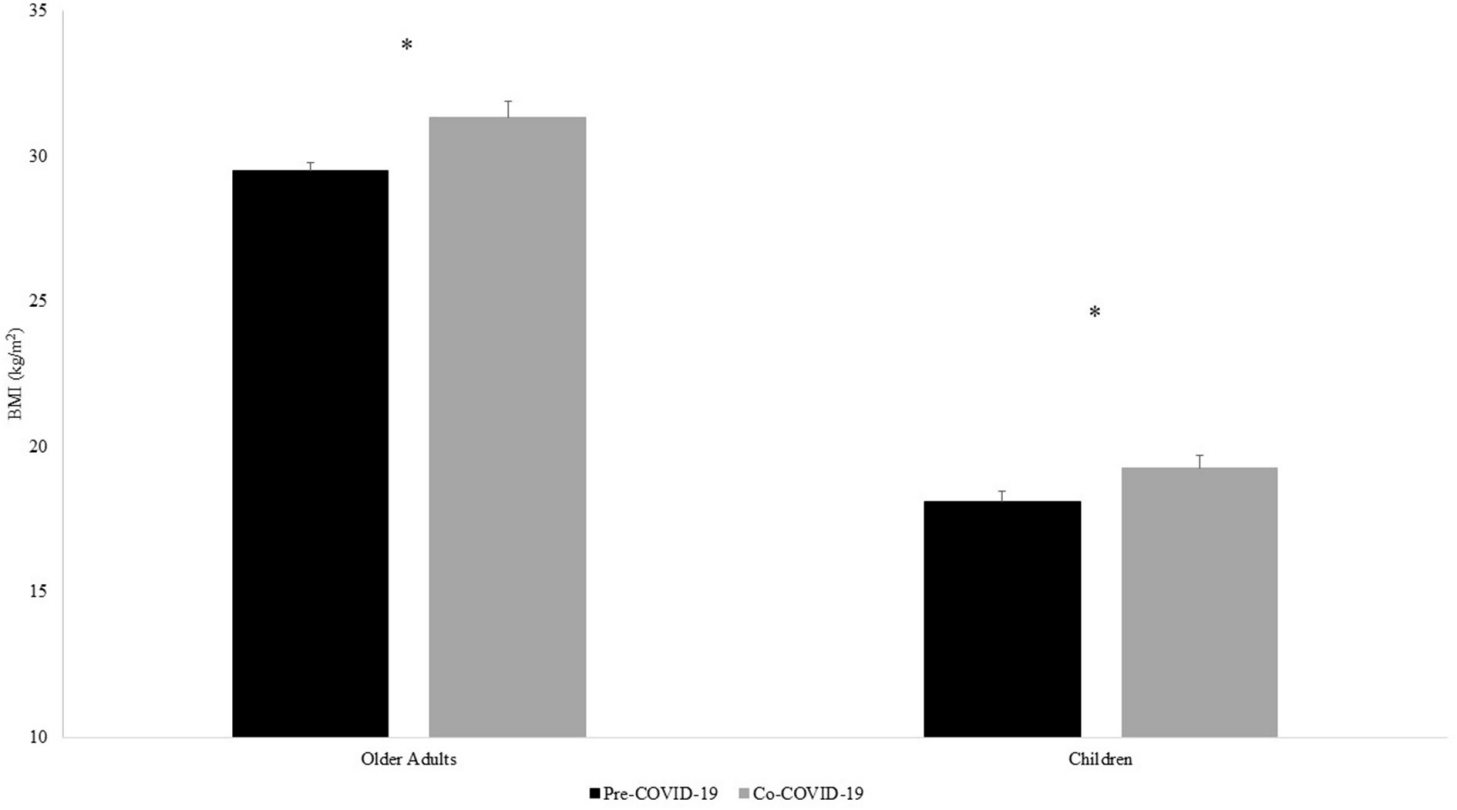
BMI (Means ± Standard Error) for Pre- and Co-COVID children and older adults. *T-test is significant at *p* ≤ 0.05.

The ANCOVA for BMI controlling for age and sex revealed differences between pre- and co-COVID older adults. Pre-COVID older adults BMI was 29.51 ± 0.26 kg/m^2^, which was significantly lower than their co-COVID counterparts (31.34 ± 0.57 kg/m^2^), *F*_(3, 589)_ = 8.44, *p* = 0.004, η^2^ = 0.014. However, this effect was non-significant after also adjusting for VO_2_max [*F*_(4, 587)_ = 0.22, *p* = 0.64, η^2^ = 0.000, see Figure 2].

### BMI in children

The ANCOVA for BMI controlling for age and sex revealed differences between pre- and co-COVID children. Pre-COVID children had a BMI of 18.13 ± 0.35 kg/m^2^, which was significantly lower than their co-COVID counterparts (19.27 ± 0.44 kg/m^2^), *F*_(3, 196)_ = 3.99, *p* = 0.05, η^2^ = 0.02, see Figure 2. Interestingly, after also adjusting for VO_2_max, the effect was not significant [*F*_(4, 195)_ = 0.39, *p* = 0.53, η^2^ = 0.002].

### BMI% in children

There were no significant differences in BMI% between pre-COVID children (57.64 ± 2.65%) and co-COVID children (63.38 ± 3.64%), *t*_(198)_ = −1.9, *p* = 0.19, CI: −14.43, 2.95.

### Regression analyses with days since shutdown

#### VO_2_max and days since shutdown in older adults

**Figure 3:**
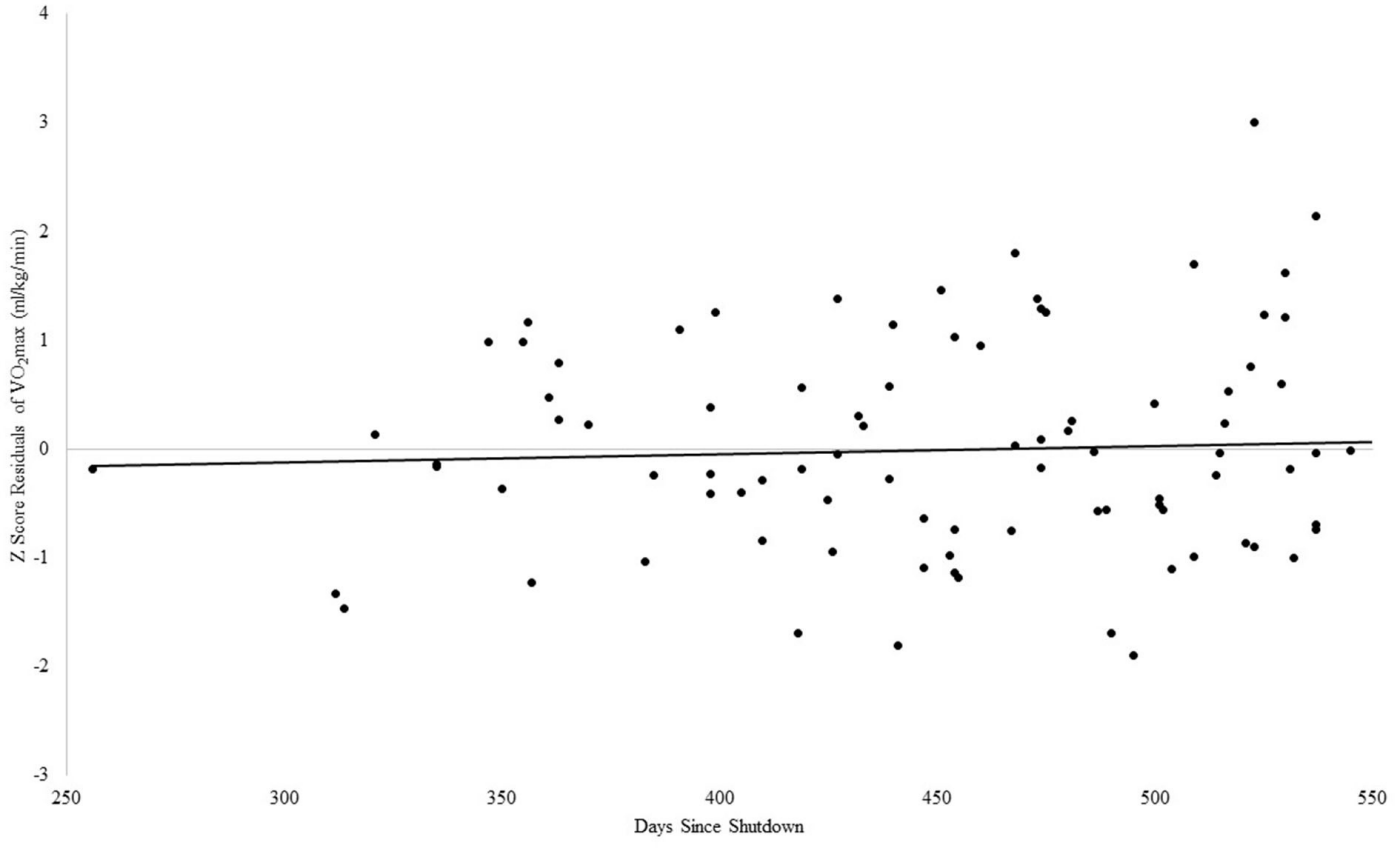
Association between days since COVID-19 shutdown and VO_2_max in older adults

For VO_2_max, the Step 1 regression analysis was significant, adjusted *R*^2^ = 0.18, *F*_(2, 97)_ = 11.86, *p* ≤ 0.001. In Step 2, the addition of days since shutdown did not account for an incremental amount of variance in VO_2_max beyond associated descriptive variables (β = 0.05, *p* = 0.58), see Figure 3.

#### VO_2_max and days since shutdown in children

**Figure 4:**
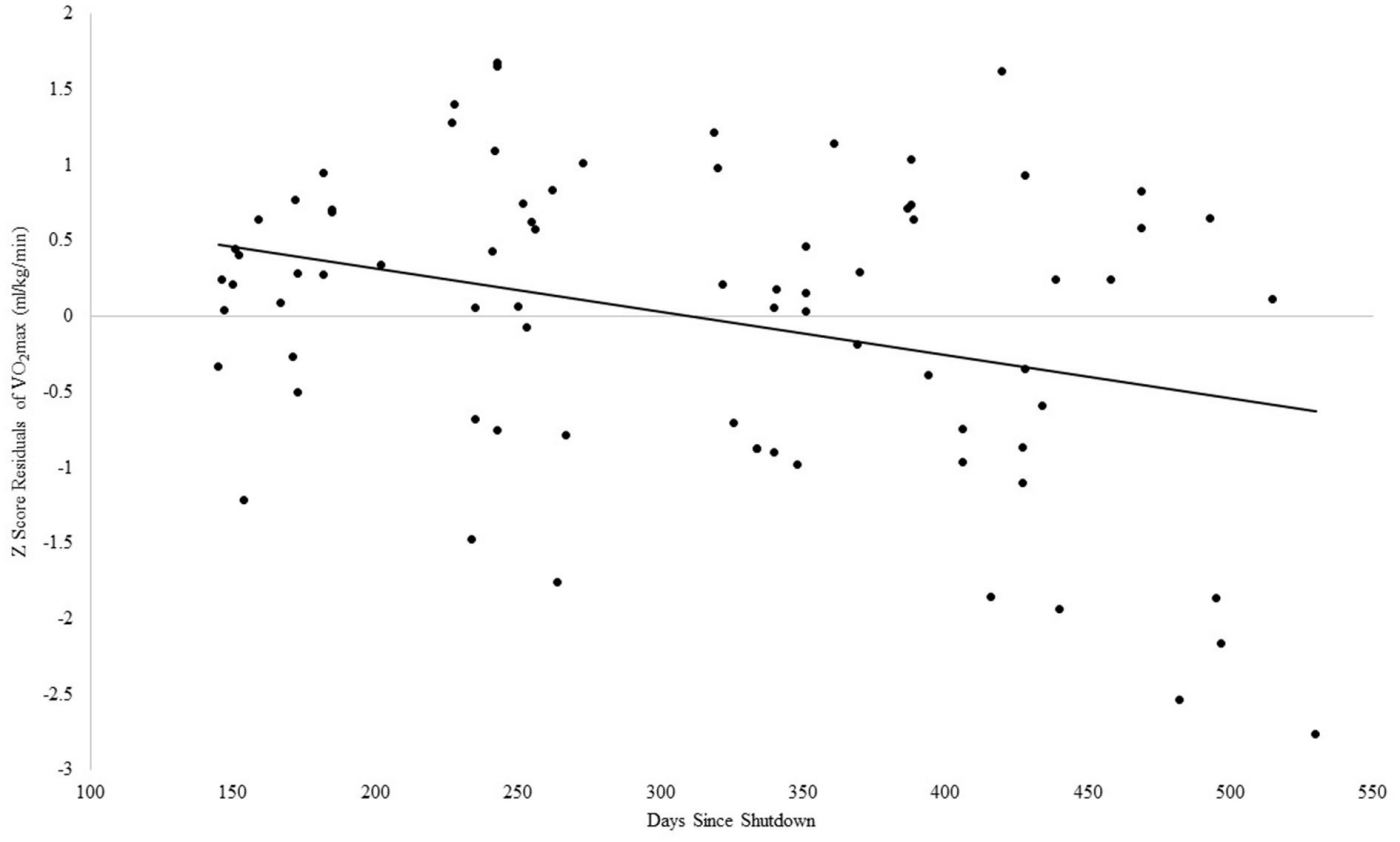
Association between days since COVID-19 shutdown and VO_2_max in children.

For VO_2_max, the Step 1 regression analysis was significant, adjusted *R*^2^ = 0.06, *F*_(2, 75)_ = 3.24, *p* = 0.04. With the addition of days since shutdown, Step 2 was also significant, Δ*R*^2^ = 0.11, *F*_(3, 74)_ = 5.86, *p* = 0.001, such that more days since the shutdown was associated with lower VO_2_max, with days since shutdown accounting for an incremental amount of variance in VO_2_max beyond the associated descriptive variables, β = −0.36, *p* = 0.002, see Figure 4.

#### BMI and days since shutdown in older adults

**Figure 5:**
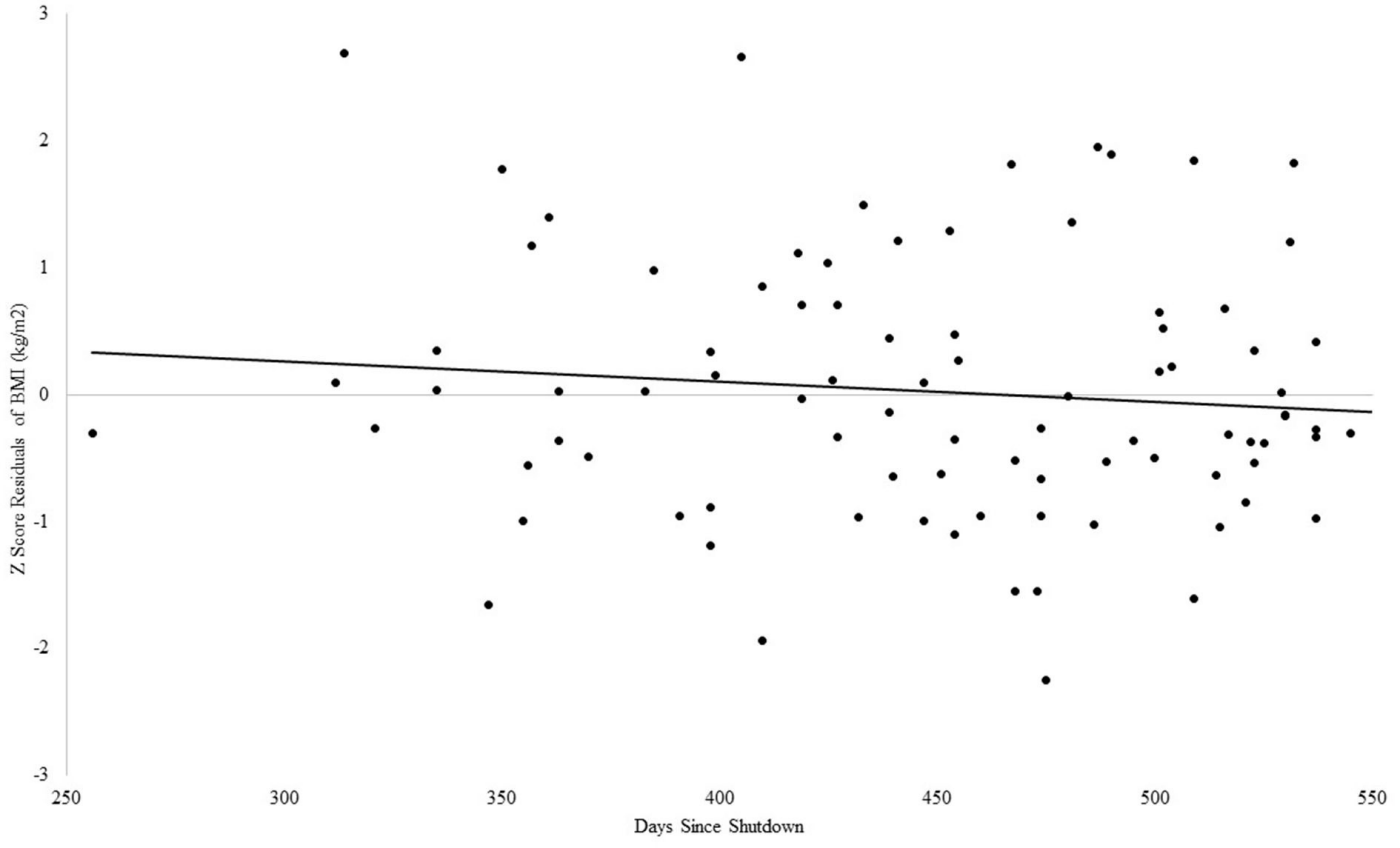
Association between days since COVID-19 shutdown and BMI in older adults.

For BMI, the Step 1 regression analysis was not significant, adjusted *R*^2^ = 0.3, *F*_(2, 97)_ = 2.49, *p* = 0.09. In Step 2, the addition of days since shutdown did not account for an incremental amount of variance in VO_2_max beyond associated descriptive variables (β = −0.12, *p* = 0.25), see Figure 5.

#### BMI and days since shutdown in children

**Figure 6:**
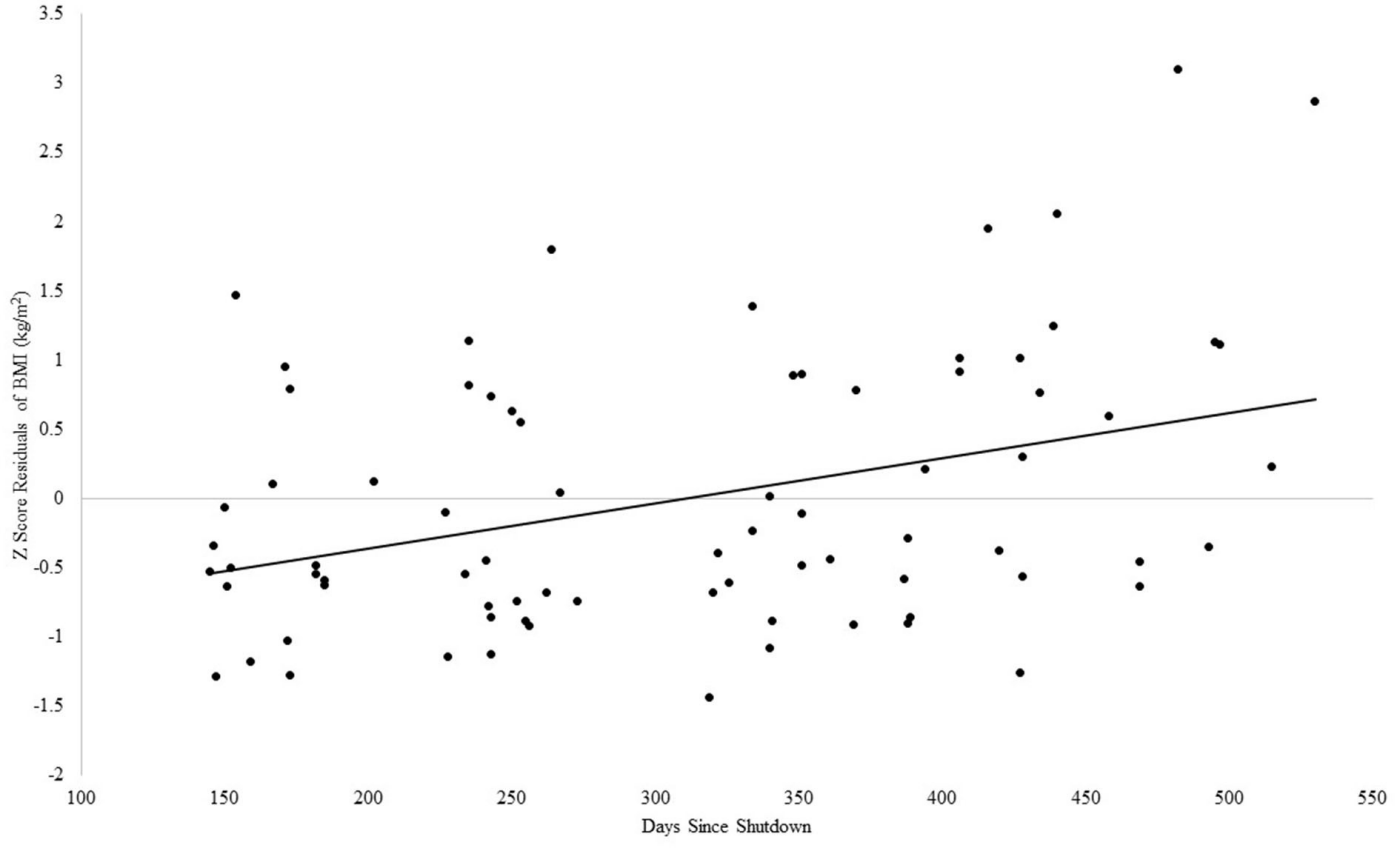
Association between days since COVID-19 shutdown and BMI in children.

For BMI, the Step 1 regression analysis was not significant, adjusted *R*^2^ = 0.01, *F*_(2, 75)_ = 1.26, *p* = 0.29. However, with the addition of days since shutdown, Step 2 was significant, Δ*R*^2^ = 0.15, *F*_(3, 74)_ = 5.66, *p* = 0.002, such that more days since the shutdown was associated with higher BMI, with days since shutdown accounting for an incremental amount of variance in BMI beyond the associated descriptive variables, β = 0.43, *p* ≤ 0.001, see Figure 6.

## Discussion

While previous investigations have examined changes in PA and BMI due to the COVID-19 pandemic, to our knowledge, this is the first study to examine differences in both CRF and BMI collectively before and following the start of the COVID-19 stay-at-home advisory in two populations particularly susceptible to the effects of COVID-19 mitigation strategies, children and older adults. Aerobic fitness levels were lower in both children and older adults following the start of the COVID-19 stay-at-home advisory. In addition, BMI was higher following the start of the COVID-19 stay-at-home advisory compared to before the stay-at-home advisory. However, these differences in BMI were not significantly different after controlling for CRF levels. Thus, our results suggest that the COVID-19 pandemic mitigation measures may have contributed to lower CRF levels in both older adults and children. Importantly, group differences in CRF remained significantly different in both older adults and children when controlling for BMI, whereas the group differences in BMI between pre and co-COVID were not significant after controlling for aerobic fitness, suggesting that the changes in CRF were relatively independent from the changes in BMI. Interestingly, these differences were exacerbated with time (i.e., days since the onset of the stay-at-home advisory) for children but not older adults.

It is worth noting that the demographics of the child participants tested before and following the start of the COVID-19 stay-at-home advisory were slightly different. However, the demographics of children tested following the start of the COVID-19 stay-at-home advisory would generally have led to greater fitness levels in children. For example, following the start of the COVID-19 stay-at-home advisory, there were more male children tested, and they were older than their pre-COVID peers. Nonetheless, our results remained significant even when controlling for differences due to age and sex.

These findings warrant immediate and significant public health concern. Across 793 participants and three geographically separate testing sites in the U.S.A., CRF was significantly lower in older adults and children when comparing participants tested following the start of the COVID-19 stay-at-home advisory to those recruited prior to the COVID-19 pandemic. The substantially lower CRF of 30–53% observed following the start of the COVID-19 stay-at-home advisory could have far-reaching and protracted health consequences.

CRF reflects the body's ability to capture, transport, and use oxygen and is dependent on many preceding and concurrent physiological processes including ventilation and diffusion, the transport of blood from the heart to match oxygen requirements, and the ability of muscle cells to use oxygen (49). Importantly, CRF rapidly declines in periods of sedentariness and inactivity, while recovery of CRF is generally slow, particularly in older adults. For example, a meta-analysis of PA trials found that older adults (~67.1 years) needed to exercise more than 3 × /week, for ~38 min, at an intensity of 40–75% of HR reserve, for nearly 20 weeks (50) to increase VO_2_max by 16.3%. In adults, research suggests that improving CRF by 1 mL/min/kg is associated with a 9% reduction in all-cause mortality (51); and when unfit individuals improve their CRF over an average of 8 years, they have a 35% lower mortality risk compared to individuals who remain unfit (52). Taken together, these findings suggest that a reduction in CRF in older adults during a period of reduced activity induced by the pandemic might require protracted recovery. The consequences of decreased activity and fitness in older adults have broad, far reaching detrimental effects that include frailty, sarcopenia, risk of falls, mental health, cardiovascular disease, and many others (19). In addition, COVID-19 usually compromises lung function and impacts major organ systems (53), and thus poor CRF may exacerbate COVID-19 outcomes.

In children, those in the lowest quartile for CRF have ~5 times greater risk of cardiovascular disease (54). Children might be particularly susceptible to long-term damage associated with low CRF because childhood fitness is predictive of both adult CRF and PA levels (55) and childhood PA is predictive of lower incidence of numerous adult onset diseases (56). As such, decreases in CRF during the COVID-19 pandemic may have serious long-term health consequences for today's youth (57, 58). Thus, approaches are urgently needed to modify the lifestyle changes induced by the pandemic to limit the negative health consequences of reduced activity.

Preliminary differences in BMI appeared in children, which is in agreement with a meta-analysis that hypothesized that adolescents and children may have been more prone to gain weight during the stay-at-home advisory period (59). These differences suggest that children may have been more physically active before the COVID-19 shutdown due to school and extracurricular activities. Additionally, the COVID-19 pandemic has been associated with an increase in the consumption of high-calorie snack foods in children (60). These findings are in agreement with other research that suggests an increase in BMI in children due to the COVID-19 pandemic (24). However, it is important to note that the differences in BMI between pre- and co-COVID children disappeared when examining percentiles and when controlling for CRF, highlighting the critical impact of CRF.

In the current sample, there were differences in BMI in older adults. Previous research suggests that changes in weight in older adults during the COVID-19 pandemic are varied. A study of 4,400 individuals aged 65 and older in Italy showed no changes in weight during the COVID-19 pandemic (31). This contrasts with data from the Netherlands (*n* = 1,119), which found an increase in body mass in elderly individuals during the pandemic (61). In addition, elderly women in Brazil reported significant increases in both weight and BMI ~1 year after the COVID-19 pandemic began (62). While we observed differences in BMI between pre- and co-COVID older adults, these effects were no longer significant when controlling for CRF, again emphasizing the important role of CRF.

These changes in weight and BMI are concerning as they stand to exacerbate the ongoing obesity epidemic. Similar to PA/fitness, obesity is related to a number of comorbidities including type-2 diabetes, cardiovascular disease, sleep disorders, and others (63). In addition, obesity is a risk factor for COVID-19 infection and severe disease (64–66). Increased BMI is one of the greatest risk factors for adverse outcomes in individuals who contract COVID-19 in both children and adults. In a large investigation of 148,494 adults diagnosed with COVID-19 during a hospital visit, over 50% had obesity. Of even greater concern, obesity was a risk factor for hospitalization, mechanical ventilation, and death (67). These consequences extend to children as well, such that children with obesity have a 3.1 times higher risk of hospitalization from COVID-19 infection, and a 1.4 times higher risk of severe illness when hospitalized (68).

The disruption from the COVID-19 shutdown resulted in a significant disruption in normal, everyday life, potentially inducing unhealthy lifestyle behavior changes. Further research and interventions are needed to monitor long-term health consequences. Interventions to address the decreases in aerobic fitness alongside increases in BMI are urgently needed. With increased BMI and lower CRF, COVID-19 may impact clinical practice for years to come. Unfortunately, the impact of the COVID-19 shutdown may also result in more people being at high risk throughout the continuation of the pandemic. Given that COVID-19 is entering the endemic phase, and instead may be a virus that we need to adapt to, in addition to other public health issues that may require stay-at-home advisories, the higher BMI observed in the present study as well as others (24, 59, 61, 62), coupled with the risk for severe infection (69, 70), warrant considerable concern and public health action.

Taken together, these findings emphasize the need to develop public health actions and clinical interventions to reduce the negative impact of COVID-19 mitigation measures on lifestyle and optimize health. Our findings are in agreement with those of Katsoulis et al. (71), who suggest that prevention of obesity and promotion of physical activity could be as important as social distancing and stay-at-home orders during the COVID-19 pandemic- both to prevent severe COVID-19 infection outcomes, as well as overall lifelong health (71). Therefore, it is critically important to create versatile physical activity opportunities, and to support children and older adults in improving their PA behaviors to modify the current unhealthy trajectories. Countermeasures to mitigate the adverse impact of the COVID-19 pandemic on body composition, CRF, and physical activity need to be innovative, robust, and highly adaptable strategies to lifestyle health and wellness.

Our findings are even more striking because we tested individuals who never tested positive for COVID-19 prior to their participation in the VO_2_max and BMI assessments, suggesting that social distancing guidelines, stay-at-home advisories, overall concern regarding the virus, and other behavior changes taken to reduce potential exposure to the virus may be associated with changes in physiological health (i.e., CRF and BMI) in ways that are not immediately evident. The considerable pandemic-related differences in CRF and BMI should be considered a major challenge for public health agencies, as it may further complicate pandemic recovery. While unintended, steps to reduce the spread of COVID-19 may have had unforeseen physiological consequences for CRF and BMI. Thus, urgent public health attention is necessary to promote physical activity with the aim of maintaining CRF levels and BMI during pandemic shutdowns.

We cannot rule out several competing explanations for the observed pandemic-related differences in CRF. Specifically, our recruitment strategies, although identical from pre-COVID to pandemic periods, may have identified participants who were characteristically different from those identified pre-pandemic (e.g., habitually less active, more sedentary, poorer diet). However, since participants were willing to visit a research setting during the pandemic, it is possible that these individuals made the least amount of change to their daily behaviors, suggesting that the observed differences may be an underestimate of the true negative consequences of the pandemic on CRF. In addition, there is a chance that some of the participants, unbeknownst to them, may have had COVID-19 and were never tested, and thus may have residual effects of the virus. Furthermore, this study is cross sectional in design, and thus causal inference cannot be assumed. Because these were separate groups of participants, there is a chance that confounders and unmeasured factors may impact the findings. These results do not contradict or eliminate the need for stay-at-home guidelines, social distancing, and other policies put into place and enforced by health officials, but rather indicate that these guidelines should be accompanied by recommendations for maintaining PA levels. This is critically important as recent research suggests that individuals who engaged in PA regularly had an 11% lower risk of getting COVID-19, and of those individuals that did contract COVID-19, they had a 34% lower risk of severe disease (72). While public health measures such as social distancing, school closures, and other non-pharmaceutical interventions were implemented to save lives (73, 74), the impact of these measures on CRF should be added to the list of unintended consequences. This is critical, as emerging evidence suggests that the decreases in PA observed during the COVID-19 pandemic have not improved with the lifting of mitigation strategies, and highlight ongoing challenges (75). As such, thoughtful measures and guidance should be provided to encourage PA to counteract these consequences. These findings have high public health relevance during necessary periods of social isolation, stay-at-home advisories, and/or closure of public facilities in the midst of a pandemic such as COVID-19.

## Data availability statement

The datasets presented in this article are not readily available because data collection is ongoing. When complete, the datasets will be available from the corresponding author on reasonable request. Requests to access the datasets should be directed to l.raine@northeastern.edu.

## Ethics statement

All participants provide written assent/consent (and legal guardians provide written informed consent) in accordance with the Institutional Review Board of Northeastern University (17-07-11 and 17-05-02), University of Pittsburgh (19110244), or University of Kansas (00140896). Informed consent was obtained from all individual participants included in the study. Informed consent was obtained from legal guardians and informed assent was obtained from child participants.

## Author contributions

All authors have contributed to the writing of the paper. All authors contributed to the article and approved the submitted version.
